# P04.75. Post-treatment hot flash severity and integrative medicine (IM) use among women with a history of breast cancer

**DOI:** 10.1186/1472-6882-12-S1-P345

**Published:** 2012-06-12

**Authors:** K Chandwani, K Mustian, J Roscoe, C Heckler, S Mohile, J Wade, J Kirshner, G Morrow

**Affiliations:** 1 University of Rochester Medical Center, Rochester, USA; 2 Medical Oncology/Hematology, Cancer Care Center of Decatur, Decatur, USA; 3 Hematology Oncology Associates of Central New York, Syracuse, USA

## Purpose

Hot flashes (HF) are common in women with breast cancer (BC) and reduce their quality of life. Currently, moderately effective pharmacologic agents are associated with bothersome side effects; the efficacy of non-pharmacologic treatments including IM, which are commonly used by cancer patients, is not exactly known. This study examined the association between HF severity in women with breast cancer at six months following the end of cancer treatment (FU) and their IM use.

## Methods

In a longitudinal study of a nationwide sample of cancer outpatients who underwent standrad treatment, women with BC who completed their treatment were included for analysis (N=373). Women rated their HF severity responding to a question about HF “at its worst” on a scale of 0 (not present) to 10 (as bad as you can imagine) and they also reported on their use of 13 IM techniques (yes/no) at FU.

## Results

HF was reported by 73% women at follow-up. Ninety-six percent were Caucasian and 4% African-American. The range of IM use (exercise, prayer, relaxation, chiropractor, massage, imagery, spirituality, diet, herbs, vitamins, group therapy, hypnosis, and acupuncture) was 2-65%. A multiple linear regression showed that HF severity was significantly associated with exercise (p=0.01) and vitamin use (p<0.0001) adjusted for significant demographic variables (age and race); more severe HF was observed in vitamin-users and those who did not do exercise. There was a significant effect of age (<55 years vs. >55 years, p<0.007), and race (p=0.001), with younger women and African-American women reporting more severe HF.

## Conclusion

The majority of participants used integrative medicine. More severe hot flashes were significantly associated with no exercise and use of vitamins. HF was more severe in younger women and African-American women; future studies are needed to corroborate the results and to ensure safe and favorable outcomes while managing HF.

